# Incomplete Penetrance and Variable Expressivity: Hallmarks in Channelopathies Associated with Sudden Cardiac Death

**DOI:** 10.3390/biology7010003

**Published:** 2017-12-26

**Authors:** Monica Coll, Alexandra Pérez-Serra, Jesus Mates, Bernat del Olmo, Marta Puigmulé, Anna Fernandez-Falgueras, Anna Iglesias, Ferran Picó, Laura Lopez, Ramon Brugada, Oscar Campuzano

**Affiliations:** 1 Cardiovascular Genetics Center, University of Girona-IDIBGI, 17190 Salt, Spain; aperez@gencardio.com (A.P.-S.); jmates@gencardio.com (J.M.); bdelolmo@gencardio.com (B.d.O.); mpuigmule@gencardio.com (M.P.); afernandez@gencardio.com (A.F.-F.); annai@brugada.org (A.I.); ferran.pico@gencardio.com (F.P.); llopez@gencardio.com (L.L.); rbrugada@idibgi.org (R.B.); 2 Centro de Investigación Biomédica en Red de Enfermedades Cardiovasculares (CIBERCV), 28029 Madrid, Spain; 3 Medical Science Department, School of Medicine, University of Girona, 17003 Girona, Spain; 4 Cardiology Service, Hospital Josep Trueta, 17003 Girona, Spain

**Keywords:** sudden cardiac death, channelopathies, genetics, incomplete penetrance, variable expressivity

## Abstract

Sudden cardiac death is defined as an unexpected decease of cardiac origin. In individuals under 35 years old, most of these deaths are due to familial arrhythmogenic syndromes of genetic origin, also known as channelopathies. These familial cardiac syndromes commonly follow an autosomal dominant pattern of inheritance. Diagnosis, however, can be difficult, mainly due to incomplete penetrance and variable expressivity, which are hallmarks in these syndromes. The clinical manifestation of these diseases can range from asymptomatic to syncope but sudden death can sometimes be the first symptom of disease. Early identification of at-risk individuals is crucial to prevent a lethal episode. In this review, we will focus on the genetic basis of channelopathies and the effect of genetic and non-genetic modifiers on their phenotypes.

## 1. Introduction

Sudden death (SD) is defined as a death that occurs unexpectedly within 1 h of the onset of symptoms or occurs un-witnessed within 24 h of the deceased being seen alive and in a normal state of health before the body is found [1]. Nearly 80% of all SDs are of cardiac origin (sudden cardiac death, SCD). SCD is the leading cause of death in the Western world accounting for an estimated 15–20% of all deaths [2]. The median age ranges between 65 and 70 years with a male prevalence [3,4].

Coronary heart disease is the predominant cause of SCD among individuals over 55 years old; in young adults less than 35 years old, arrhythmogenic syndromes (cardiomyopathies and channelopathies) are the main cause of SCD [5]. Many of these cardiac causes of SCD among children and young adults have an underlying genetic basis, defined by monogenic genetic causes and are therefore inherited. The incidence of SCD in infants and youth less than 35 years old is approximately 2 to 8/100,000 person years, accounting for 2–10% of adult SCD [3,4].

In this review, we will focus on cardiac channelopathies, electrical disorders in structurally normal hearts caused by pathogenic alterations in genes encoding cardiac ion channels or their regulatory proteins.

## 2. Channelopathies

Inherited arrhythmogenic diseases are the main cause of SCD in young individuals. Unfortunately, SCD can be the first manifestation of one of these diseases; thus, identifying these disorders is crucial for family members. Consequently, current guidelines recommend that, for all victims having a structurally normal heart but no conclusive cause of death, a complete autopsy be performed, including molecular investigation to rule out a cardiac channelopathy [6].

### 2.1. Long QT Syndrome

Long QT syndrome (LQTS) is an inherited arrhythmic disorder characterized by a QT interval prolongation in the ECG; QTc is prolonged if >440 ms in men or >460 ms in women, in a structurally normal heart. This entity may be acquired (mainly due to pharmacological effects) or congenital. Electrical alterations may lead to palpitations, syncope and even SCD [7]. LQTS is the most prevalent inherited cardiac arrhythmia with an estimated prevalence of 1:2000–1:5000 and the leading cause for SD in young people [8].

LQTS is mainly transmitted in an autosomal dominant pattern (Romano–Ward syndrome); however, a few cases may follow an autosomal recessive pattern (Jervell and Lange–Nielsen syndrome) [9].

Current guidelines recommend genetic analysis of the three main prevalent LQTS-associated genes: *KCNQ1* (encoding the Kv7.1, 30–35% of cases), *KCNH2* (encoding the Kv11.1 potassium channel, 25–30% of cases) and *SCN5A* (encoding the Nav1.5 sodium channel, 5–10% of cases) [10]. Nevertheless, a few pathogenic variants have been identified in an additional 14 genes following an autosomal-dominant pattern of inheritance (*AKAP9*, *ANK2*, *CACNA1C*, *CALM1*, *CALM2*, *CALM3*, *CAV3*, *KCNE2*, *KCNJ2*, *KCNJ5*, *RYR2*, *SCN1B*, *SCN4B* and *SNTA1*), in another gene following an autosomal-recessive pattern (*TRDN*) and in one gene following both autosomal-dominant and recessive patterns of inheritance (*KCNE1*) [11].

Pathogenic genetic variants identified in potassium genes lead to a loss of function [5] whereas mutation in sodium and calcium channels lead to gain of function effect [12].

### 2.2. Brugada Syndrome

Brugada Syndrome (BrS) is a rare arrhythmogenic disease (estimated prevalence of 1–5/10,000 inhabitants) characterized by a ST-segment elevation in leads V1–V3 and cardiac conduction abnormalities in patients without structural heart alterations, leading to a high risk for ventricular arrhythmias and SCD [7].

BrS typically manifests in the third or fourth decade of life and mainly in men who are at rest. BrS shows a lower prevalence in Northern Europe (1.1 in 100,000 in Denmark) [13] and seems to be endemic in South-Eastern Asia, where the prevalence is 15–20 in 10,000 inhabitants [14].

This syndrome is inherited following an autosomal pattern. Nearly 450 pathogenic variants in 25genes have been associated with BrS (*ABCC9*, *CACNA1C*, *CACNA2D1*, *CACNB2b*, *FGF12*, *GPD1-L*, *HCN4*, *HEY2*, *KCND2*, *KCND3*, *KCNE3*, *KCNE5*, *KCNH2*, *KCNJ8*, *LRRC10*, *PKP2*, *RANGRF*, *SCN10A*, *SCN1B*, *SCN2B*, *SCN3B*, *SCN5A*, *SEMA3A*, *SLMAP* and *TRPM4*). Variants in these genes can explain 30–35% of the cases, thus 65–70% of BrS cases remain genetically unsolved [11]. These genes encode for sodium, potassium and calcium ion channels or associated proteins. Despite recent genetic reports identifying patients with variants in these genes, conclusive association between most of these genes and BrS remains to be clarified. Current clinical guidelines only recommend genetic analysis of *SCN5A* (encoding the Nav1.5 sodium channel) since it is attributable in 25% of all BrS cases [6]. Loss-of function mutations in the SCN5A-encoded a-subunit of the cardiac sodium channel represent the most common genetic substrate for BrS cases. Although most of the genetic mutations in the genes involved in BrS result in a loss-of function effect, variants in *KCND3*, *KCNE3* and *KCNE5* genes cause a gain-of function effect in BrS patients.

### 2.3. Catecholaminergic Polymorphic Ventricular Tachycardia

Catecholaminergic polymorphic ventricular tachycardia (CPVT) is an inherited disease characterized by the presence of adrenergic-induced bidirectional or polymorphic ventricular tachycardia in individuals with a normal basal electrocardiogram and structurally normal heart. CPVT patients can present syncope and SCD triggered by physical or emotional stress at young ages and SCD can be the first manifestation in up to 30% of cases [15].

The prevalence of CPVT in the general population is estimated to be 1:10,000. Even though it is a rare entity, timely recognition is of vital importance because CPVT plays an important role in SCD in the young [16].

Sixty to sixty-five percent of CPVT patients carry a pathogenic variant in the *RYR2* gene (encoding the ryanodine receptor 2), which is transmitted in an autosomal dominant pattern. Other genes have also been associated with CPVT (*ANK2*, *CALM1*, *CALM2*, *CALM3*, *CASQ*2, *KCNJ2* and *TRDN*). CPVT is rarely diagnosed with a pathogenic variant in any of these other genes [10,17]. Pathogenic variants in the *CASQ2* gene are transmitted following an autosomal recessive pattern but are also found in a small percentage of cases.

### 2.4. Short QT Syndrome

Short QT syndrome (SQTS) was first described in 2000 by Gussak et al. as a new clinical entity [18]. This syndrome is extremely rare with an estimated prevalence of less than 1 in 10,000 [19] and characterized by abnormally short QT intervals on the ECG. Currently, SQTS is defined as a QTc ≤ 330 ms or a QTc interval <360 ms and one or more of the following: history of cardiac arrest or syncope, family history of SCD at age 40 or younger, or a family history of SQTS [20].

The disease appears to be highly lethal in all age groups, including children in their first months of life and the probability of a first cardiac arrest by the age of 40 years is >40% [6].

Currently, six genes encoding potassium and calcium channels have been associated with SQTS, following an autosomal dominant pattern of inheritance: *KCNH2*, *KCNQ1*, *KCNJ1*, *CACNA1C*, *CACNB2* and *CACNA2D1*. A comprehensive genetic analysis identifies the genetic origin of this entity in nearly 60% of cases. Clinical guidelines recommend performing a genetic analysis of the main genes: *KCNH2* and *KCNQ1* [6].

## 3. Incomplete Penetrance and Variable Expressivity

Generally, the inheritable cardiac arrhythmia syndromes follow an autosomal dominant pattern of inheritance but may exhibit incomplete penetrance, variable expressivity and phenotypic overlap.

**Figure 1:**
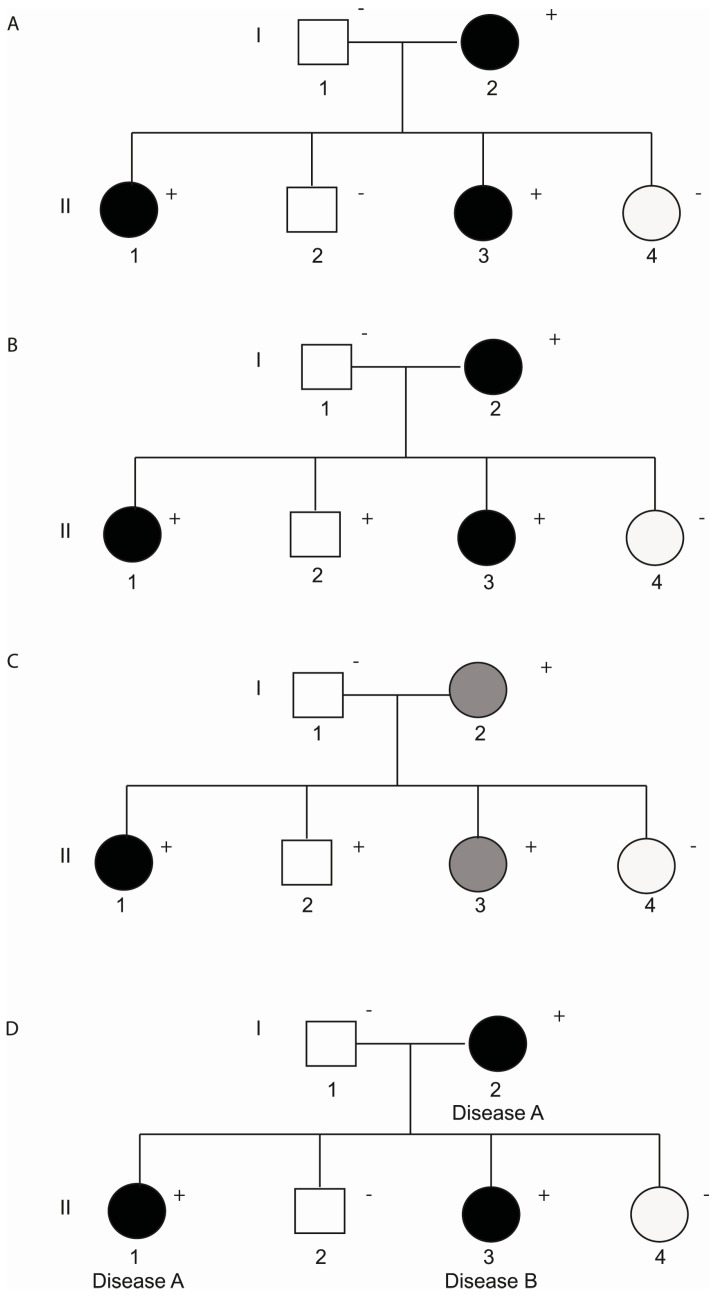
(**A**) Pedigree displaying complete penetrance; (**B**) Pedigree with incomplete penetrance because individual II:2 carries the mutation but is not affected; (**C**) Pedigree demonstrating incomplete penetrance and variable expressivity due to individual II:3 who carries the mutation but presents a less severe disease; (**D**) Pedigree showing phenotypic overlap because individual II:3 shows a different cardiac channelopathy. Clinically affected patients are shows in black, clinically unaffected patients are show in white and grey indicates a less severe phenotype. A plus sign indicates a carrier of the genetic variant. A minus sign indicates a non-carrier.

Penetrance is defined as the proportion of people with a specific genotype who manifest a particular clinical characteristic or phenotype. Variable expressivity refers to the series of signs and symptoms that can occur in different people with the same genetic condition. Both incomplete penetrance and variable expressivity are probably due to a combination of genetic, environmental and lifestyle factors. Only a few studies have focused on these modifiers, probably due to the high number of parameters that must be analysed. In addition to incomplete penetrance and variable expressivity, some cases with disease-causing mutations display phenotypic overlap, also known as pleiotropy. The pleiotropy occurs when mutations in a single gene may have a different effect and result in different heritable cardiac channelopathies in the same multigenerational pedigree (Figure 1).

Mutations in specific genes have been associated with different hereditary cardiac arrhythmias. For instance, mutations in *SCN5A* have been related to LQTS, BrS, sick sinus syndrome and cardiac conduction disturbance, leading to overlapping electrocardiographic phenotypes. In 2016, Veltmann et al. demonstrated high penetrance for LQTS, BrS and cardiac conduction disease in a large family harbouring the *SCN5A*-E1784K mutation [21]. There are several other *SCN5A* mutations, including delK1500-*SCN5A*, delKPQ1505-1507 and 1795insD-*SCN5A*, associated with a spectrum of arrhythmia phenotypes [22].

Distinct overlapping features have been identified in BrS and arrhythmogenic right ventricular cardiomyopathy (ARVC) patients and this has been a subject of intense debate. Both syndromes could interact at a genetic level, considering that mutations in genes linked with ARVC could cause electrophysiological changes that facilitate the pathophysiological mechanism of BrS [23].

Another explanation for the phenotypic overlap could be a misdiagnosis of the heritable cardiac channelopathy phenotypes. The intravenous flecainide test allows unmasking BrS cases and oral flecainide is a proposed treatment for LQTS3 patients. In 2000, Priori et al. demonstrated that flecainide may induce an elevation of the ST segment in the ECG in patients with LQTS3, leading to a misdiagnosis of these cases [24].

The degree of penetrance among channelopathies is highly variable and also varies with the number of cases analysed among publications. In 2000, Priori et al. analysed 4 families affected by BrS based on their ECG analysis and identified a penetrance of 16% (range 12.5% to 50%) [25]. The penetrance of CPVT ranges from 63 to 78% [26,27] and for LQTS the mean penetrance is ~40% [22]. 

Huge inter-individual variability cannot be exclusively explained by a single genetic variant, which leads us to consider possible non-genetic (demographic variables, such as sex or age and exogenous factors) and genetic (coding or non-coding variants) modifiers (Table 1).

### 3.1. Non-Genetic Modifiers

Sex plays an important role in channelopathies. Males have a worse prognosis and greater risk of SCD than females. In the BrS-associated phenotype, intrinsic modifications in ionic currents and hormonal effects are thought to cause these variations [28]. Similar to this arrhythmogenic disease, SQTS, CPVT and LQTS display a sex-dependent penetrance [29,30].

Early manifestations of the symptoms may indicate an aggressive form of a disease, emphasizing the importance of changes in hormones, such testosterone [31]. On the other hand, there is some experimental evidence showing that sodium channels may decline with age leading to a more serious phenotype for a *SCN5A* pathogenic variant [32]. Mac Millant et al. identified the possibility of an age-related penetrance to the ajmaline provocation test and supports the need to retest with a negative ajmaline test in earlier childhood [33]. Conte et al. report for the first time the changes in the response to ajmaline over time and unmasked BrS in 23% of relatives with a previously negative drug test performed during childhood [34].

Several exogenous factors together with disease-causing variants can modulate ECG parameters altering the genotype-phenotype correlation. For example, the ECG pattern type I in BrS may be unmasked by fever, excessive alcohol intake and large meals. Drugs or electrolyte imbalances in LQTS may prolong or unmask the QT interval [6].

### 3.2. Genetic Modifiers

#### 3.2.1. Coding Variants

• Rare variants

Genetic variants with minor allele frequency (MAF) <1% are considered rare and are often the main cause of heritable cardiac arrhythmias. Nevertheless, a wide percentage of families with a well-studied disease-causing mutation do not show full penetrance and complete expressivity. This fact may be due to the additive effect of multiple independent mutations, which can disturb and often increase the severity of the phenotype. More recently, it has become clear that LQTS patients carrying two or more pathogenic variants either in the same gene (compound heterozygotes) or in different genes (digenic heterozygosity) make up 4–9% of patients [35]. As a further example, a family affected by BrS was found to have a double pathogenic variant in the *SCN5A* gene associated with the severity of the disorder in the proband and his deceased sibling [36].

In 2011, the publication of exome data from the NHLBI GO Exome Sequencing project (ESP) increased access to the distribution of genetic variations in the general population. Two studies identified a high prevalence of genetic variants previously associated with LQTS (1:31) and BrS (1:23) in the new exome data [37,38]. These results suggest that some variants previously associated with a rare disease may not be disease-causing mutations.

Other types of variants, not identifiable by Sanger, can now be detected with high throughput technologies (next-generation sequencing, NGS). These include copies of large genomic regions, also known as copy number variants (CNVs). These genomic imbalances have been identified in some channelopathies, including LQTS [39] and BrS [40] but do not seem to be major contributors to these arrhythmogenic disorders (less than 5% of all diagnosed cases).

Lastly, pathogenic coding variants in genes not previously associated with the heritable cardiac arrhythmias may explain the lack of full penetrance. Whole exome sequencing in well studied families is emerging as the most feasible strategy to identify potential new arrhythmia-associated genes.

• Common variants

Variants with MAF > 1% are considered common variants or polymorphisms. A further explanation for the low penetrance in the heritable cardiac diseases is the coexistence of modified variants in the same ion channel genes, possibly common single polymorphisms, altering susceptibility to arrhythmias. These common variants could alter the severity of the disease caused by another disease-causing mutation or act independently to modulate susceptibility to ECG abnormalities in healthy individuals.

The common polymorphisms or “second hits” are located on the opposite allele of the gene carrying the primary disease-causing mutation or in a completely separate channelopathy-susceptibility gene and they can either worsen or attenuate the effect of a primary disease-causing mutation [22]. Probably one of the most studied modifiers, p.H558R_*SCN5A* (MAF = 0.2217) seems to reduce the pathogenic effect in carriers of other disease-causing mutations, producing a less severe phenotype. In 2005, Crotti et al. demonstrated that the p.K897T_*KCNH2* (MAF = 0.1871) polymorphism can act as a genetic modifier of LQTS clinical severity in the presence of other rare variants in the same gene. An additional recognized modifier of penetrance and expressivity, p.D85N_*KCNE1* (MAF = 0.009158) causes loss-of-function effects on both IKr and IKs and behaves as a disease-causing variant [41]. Moreover, these common polymorphisms occur more frequently in patients with some cardiac channelopathies than in healthy individuals [41].

Genome-wide genetic association studies (GWAS) use a statistically powerful approach to detect genetic variants with small effect sizes. GWAS studies revealed a strong and replicable association between common variants in *NOS1AP* (not previously associated with cardiac electrophysiology), *KCNQ1*, *KCNE1*, *KCNH2*, *SCN5A* and *KCNJ2* genes and QT interval duration in a control population. Another important GWAS study in BrS patients performed by Bezzina et al. in 2013 identified a significant association with the *SCN10A* locus (rs10428132) and *HEY2* gene (rs9388451). The association signals at *SCN5A-SCN10A* demonstrated that genetic polymorphisms modulating cardiac conduction can also influence susceptibility to cardiac arrhythmia. These results indicate that common genetic variation can have a strong impact on the predisposition to rare diseases [42].

#### 3.2.2. Non-Coding Variants

Only a small part of the genome encodes for proteins and at least some non-coding DNA has important biological functions. Non-coding regions located upstream (5′UTR) and downstream (3′UTR) of genes are transcribed but not translated into proteins. They have a regulatory role in gene expression, especially in the control of mRNA stability and translation. Therefore, variable transcription of different genes encoding ion cardiac channels is a possible mechanism for determining arrhythmia susceptibility.

In 2012, Amin et al. identified 3′UTR SNPs (rs2519184, rs8234 and rs10798) that potently modify the disease severity of LQT1. Patients with the derived SNP variants in their mutated *KCNQ1* allele had shorter QTc and fewer symptoms, while patients with the derived SNP variants in their normal *KCNQ1* allele had longer QTc and more symptoms [43]. Nevertheless, Crotti et al. (2016) analysed the effect of 3 mutations (rather than the 33 used by Amin et al.) in 747 subjects (versus the 168 used by Amin et al.) and demonstrated that 3′UTR SNPs are not acting as genetic modifiers in LQT1 patients [44].

MicroRNAs are small (about 22-nucleotide long) non-coding RNAs that regulate gene expression through translational repression or mRNA degradation, usually by binding to target sites located in the 3’UTRs of mRNAs. They are involved in the pathogenesis of many diseases, including cardiovascular and neurodegenerative diseases, immune disorders and cancer [45]; however, little is known about the role of miRNAs in regulating genes encoding ionic cardiac channels and may represent an important area for future analyses.

## 4. Conclusions

Cardiac channelopathies mainly follow an autosomal dominant pattern of inheritance but usually display incomplete penetrance, variable expressivity and phenotypic overlap. These phenotypic variations are due to genetic modifiers and environmental and lifestyle factors but most pathophysiological pathways implicated in these effects remain unknown. Basic analysis and further genotype-phenotype correlation studies in large families are crucial for understanding the cellular mechanism underlying these conditions as well as performing accurate SC-risk prediction for each individual.

## Figures and Tables

**Table 1:** Genetic and non-genetic factors associated with incomplete penetrance and variable expressivity.

Non-Genetic Modifiers	
Gender	Worse prognosis in males
Age	Severe phenotypes in early manifestations, age-related penetrance to the ajmaline provocation test
Exogenous factors	Fever, excessive alcohol and large meals
**Genetic Modifiers**	
Coding variants—Rare Variants (MAF < 1%)	Additive effect of multiple independent mutations
	CNVs
	New genes involved with the disease
Coding variants—Common Variants (MAF > 1%)	Second hits (i.e., p.H558R_*SCN5A*; p.K897T_*KCNH2;* p.D85N_*KCNE1*
Non-coding variants	5′UTR and 3′UTR variants, microRNAs
